# Flexible ureteroscopy with a flexible and navigable suction ureteral access sheath versus mini‐percutaneous nephrolithotomy for 1–2 cm lower pole renal stones: Protocol for an international, multicentre, randomized non‐inferiority trial (FLAME trial)

**DOI:** 10.1002/bco2.70114

**Published:** 2025-11-29

**Authors:** Wei Zhu, Jinghua Zhong, Kefeng Wang, Guangming Yin, Chi‐Ho Leung, Nariman Gadzhiev, Mehmet Ilker Gökce, Jaisukh Kalathia, Vineet Gauhar, Guangyuan Zhang, Gang Wu, Mingyong Li, Li Fang, Shaohua Zeng, Peide Bai, Ji Li, Zhenhua Zhao, Ming Xi, Xin Mai, Xianzhong Duan, Kehua Jiang, Jianwei Cao, Xianghu Meng, Shusheng Liu, Aram Aloyan, Peng Yuan, Xiaorui Zhu, Ting Huang, Jianhui Xing, Jingzeng Du, Wenqi Wu, Jinxiang Ma, Wen Zhong, Zhijian Zhao, Yongda Liu, Chi Fai Ng, Jean de la Rosette, Bhaskar Somani, Shiyong Qi, Guohua Zeng, Steffi Kar Kei Yuen

**Affiliations:** ^1^ Department of Urology and Guangdong Provincial Key Laboratory of Urological Diseases The First Affiliated Hospital of Guangzhou Medical University Guangzhou China; ^2^ Department of Urology Shengjing Hospital of China Medical University Shenyang China; ^3^ Department of Urology, The Third Xiangya Hospital Central South University Hunan China; ^4^ SH Ho Urology Centre, Department of Surgery The Chinese University of Hong Kong Hong Kong China; ^5^ Department of Urology Saint Petersburg State University Hospital St. Petersburg Russian Federation; ^6^ Department of Urology Ankara University School of Medicine Ankara Turkey; ^7^ Department of Urology and Kidney Transplantation Fortune Urology Clinic India; ^8^ Department of Urology Ng Teng Fong General Hospital Singapore Singapore; ^9^ Asian Institute of Nephrourology AINU India; ^10^ Department of Urology, Zhongda Hospital Southeast University Nanjing Jiangsu Province China; ^11^ Department of Urology, Tongji Hospital, School of Medicine Tongji University Shanghai China; ^12^ Department of Urology, The First Affiliated Hospital, Hengyang Medical School University of South China Hengyang Hunan China; ^13^ Department of Urology The First Affiliated Hospital of Ningbo University Ningbo China; ^14^ Department of Urology The Affiliated Qingyuan Hospital (Qingyuan People's Hospital), Guangzhou Medical University Qingyuan China; ^15^ Department of Urology The First Affiliated Hospital of Xiamen University, School of Medicine, Xiamen University Xiamen Fujian China; ^16^ The People's Hospital of Dehong Prefecture Dehong Yunnan China; ^17^ Department of Urology The Sixth Affiliated Hospital, School of Medicine, South China University of Technology Foshan Guangdong China; ^18^ Department of Urology Huadu District People's Hospital Guangzhou China; ^19^ Department of Urology Jiangmen Central Hospital Guangdong China; ^20^ Department of Urology Baoshan No. 2 People's Hospital Baoshan China; ^21^ Department of Urology Guizhou Provincial People's Hospital Guizhou China; ^22^ Xinhua Hospital Affiliated to Shanghai Jiao Tong University School of Medicine Shanghai China; ^23^ Department of Urology First Affiliated Hospital with Nanjing Medical University Nanjing China; ^24^ Department of Urology The Second Affiliated Hospital of Guangzhou Medical University Guangzhou China; ^25^ School of Public Health Guangzhou Medical University Guangzhou China; ^26^ International School of Medicine Istanbul Medipol University Istanbul Turkey; ^27^ Bashkir State Medical University Ufa Russia; ^28^ Department of Urology University Hospital Southampton, NHS Trust Southampton UK; ^29^ Department of Urology, Tianjin Institute of Urology The Second Hospital of Tianjin Medical University Tianjin China

**Keywords:** FANS, flexible and navigable suction ureteral access sheath, flexible ureteroscopy, lower pole, PCNL, renal stones

## Abstract

**Background:**

Lower pole renal stones measuring 1–2 cm remain challenging to treat. While mini‐percutaneous nephrolithotomy (mini‐PCNL) provides high stone‐free rates (SFRs), it carries tract‐related morbidity. Flexible ureteroscopy (f‐URS) is less invasive but limited in SFR. The flexible and navigable suction ureteral access sheath (FANS) has shown promise in improving stone evacuation and intrarenal pressure control. We hypothesize that FANS f‐URS is non‐inferior to mini‐PCNL for patients with 1–2 cm lower pole stones in SFR.

**Study Design:**

The FLAME trial is an international, multicentre, randomized, non‐inferiority study directly comparing FANS‐f‐URS with mini‐PCNL in this setting.

**Endpoints:**

The primary outcome is immediate SFR within 72 hours on low‐dose CT. Secondary outcomes include SFR at 1 month, operative time, postoperative pain, hospital stay, complications (Clavien–Dindo) and quality‐of‐life changes.

**Patients and Methods:**

A total of 640 adults with CT‐confirmed 1–2 cm lower pole renal stones will be randomized 1:1 to undergo FANS‐f‐URS or mini‐PCNL across 20 high‐volume urology centres worldwide. Randomization is centralized and stratified by site. Radiologists and statisticians will remain blinded to allocation. Sample size was calculated assuming an 85% SFR for both arms, an 8.5% non‐inferiority margin, 80% power and 15% attrition. Analyses will follow both intention‐to‐treat and per‐protocol principles.

**Trial registration:**

ClinicalTrials.gov NCT07159035.

## 1 BACKGROUND

Lower pole renal stones measuring 1–2 cm pose a unique endoscopic challenge, as there is currently no clear indication or contraindication favouring one treatment modality over the other. The latest European Association of Urology guidelines^1^ advise that, in the presence of unfavourable lower pole anatomy, “endourology” should be the first‐line approach. However, it is unclear whether flexible ureteroscopy (f‐URS) or percutaneous nephrolithotomy (PCNL) is preferred. Mini‐PCNL achieves high stone‐free rates (SFRs) but carries a greater risk of bleeding and tract‐related morbidity.^2^ In contrast, conventional f‐URS offers a minimally invasive alternative yet often results in lower SFRs in the dependent lower calyx due to difficult deflection angles, resulting in suboptimal access and fragment evacuation.^3^, ^4^

In recent years, f‐URS technology has made monumental advances. The advent of the flexible and navigable suction ureteral access sheath (FANS) and enhanced single‐use digital ureteroscope deflection mechanics allows navigation of the FANS and ureteroscope tip into target calyces that were previously challenging to reach, mitigating the traditional disadvantages of retrograde access.^5^ Current FANS and flexible ureteroscope technology have brought about active fragment evacuation, markedly enhanced intraoperative visibility, reducing intrarenal pressure and facilitating complete stone clearance in the current suction endourology era.^6^, ^7^

Given these technological improvements, several observational studies have reported favourable outcomes for FANS f‐URS in managing lower pole stones.^4^, ^8^ Currently, there is still a paucity of high‐level evidence to determine whether FANS f‐URS can achieve comparable efficacy to mini‐PCNL. With the aim of addressing this evidence gap and potentially expanding the armamentarium for intermediate‐sized renal calculi, we have designed an international, multicentre, randomized non‐inferiority trial comparing FANS f‐URS with mini‐PCNL in this challenging cohort. This study is titled the FLAME trial, an acronym for: Flexible ureteroscopy for Lower pole stones And Mini pErcutaneous nephrolithotomy.

## 2 STUDY DESIGN

The aim of this international, multicentre, randomized non‐inferiority trial is to determine whether FANS f‐URS is non‐inferior to mini‐PCNL in terms of SFR for 1–2 cm lower pole renal stones. The primary research question is whether FANS f‐URS achieves SFRs comparable to those of mini‐PCNL, and secondary objectives include comparing perioperative safety (complication rates), operative time, hospital stay duration, patient‐reported pain and recovery metrics and overall health‐related quality of life after each procedure. By evaluating both efficacy and patient‐reported outcomes, this study seeks to inform best practices for managing intermediate‐sized lower‐pole stones.

## 3 ENDPOINTS

### 3.1 Primary outcome

**Immediately stone‐free rate (SFR)**: no residual stone or stone fragments larger than 2 mm on CT scans within postoperative 72 hours is defined as stone‐free.

Residual fragments (RFs) were classified based on the bone window of CT imaging as follows:

- Grade A: 100% stone‐free/zero fragment rate (ZFR).
- Grade B: Single RF < 2 mm.
- Grade C: Single RF measuring 2–4 mm.
- Grade D: Single or multiple RF > 4 mm.

For this study, Grades A and B patients were considered stone‐free, requiring no additional imaging. Patients classified as Grades C and D were categorized as non‐stone‐free. This definition aligns with contemporary endourology trial standards.

### 3.2 Secondary outcomes

1. **Final SFR**: SFR at 1 month based on low‐dose CT scan. No residual stone, or no stone fragments larger than 2 mm under CT scan are defined as stone‐free.
2. **Operative time**: For the FANS f‐URS group, the operative time is characterized as the duration from the insertion of the endoscope into the urethra to the completion of stent placement. For the mini‐PCNL group, the operation time is defined as from retrograde placement of the ureteric catheter to the placement of nephrostomy tubing or removal of percutaneous access.
3. ***Postoperative pain levels:** assessed within 24 hours using a 10‐point visual analogue scale (VAS)*.^9^
4. **Duration of hospital stay**: *Calculated from day of surgery to day of discharge, rounded up to the nearest whole day*.
5. **Complications:** All adverse events occurring within 1 month post‐procedure, graded by the Clavien–Dindo classification.^10^
6. **Improvement of Quality‐of‐Life Score**: *Measured preoperatively and at 1 month using the Wisconsin Stone Quality of Life questionnaire*.^14^, ^16^

## 4 ELIGIBILITY CRITERIA

Inclusion criteria

1. Adults aged 18–75 years;
2. American Society of Anesthesiology (ASA) score 1–3;
3. Lower pole renal stones—single or multiple—with a maximal diameter of 1–2 cm confirmed by CT;
4. Ability to provide written informed consent and adhere to trial requirements.

Exclusion Criteria

1. Significant urinary tract anatomical anomalies (e.g. horseshoe kidney, ileal conduit);
2. Stones located within a calyceal diverticulum;
3. History of open nephrolithotomy or ureterolithomy (due to resultant intrarenal anatomical distortion);
4. Absolute contraindications to either FANS‐f‐URS or mini‐PCNL, including:
  - Uncorrectable coagulopathy;
  - Active, uncontrolled urinary tract infection;
  - Severe cardiopulmonary disease precluding safe general anaesthesia (e.g. decompensated heart failure, refractory COPD);
  - Pregnancy;
  - Inability to tolerate lithotomy or prone positioning;
5. Inability to understand or complete trial documentation.

## 5 METHODS AND ANALYSIS

### 5.1 Study overview

This is a multicentre, prospective, non‐inferiority, randomized controlled trial. The study will be conducted in accordance with the Declaration of Helsinki and the International Conference on Harmonization – Good Clinical Practice (ICH‐GCP).^11^

### 5.2 Patient and public involvement

Two patient representatives reviewed the consent form and provided feedback on language clarity. There is no other direct patient involvement in trial design.

### 5.3 Setting

This international trial will be conducted at 20 high‐volume urology centres—17 in China, one in the Russian Federation, one in India, and one in Turkey—each with established expertise in renal stone management. Each participating site performs a minimum of 300 FANS f‐URS and 200 mini‐PCNL procedures annually, ensuring both proficiency and timely patient enrolment.

### 5.4 Identification and enrolment of potential participants

Procedures for identifying and consenting participants will be tailored to each centre's local practices and patients' needs. Clinicians or trained research staff will screen all patients presenting with suspected renal stones during routine workflow. A detailed screening log will record every assessed patient and the reason for exclusion (e.g., ineligibility, refusal), to populate the CONSORT flow diagram.

When a 1–2 cm lower pole stone is confirmed by CT and all eligibility criteria are met, patients will receive an information leaflet describing the trial's purpose, procedures, potential benefits and risks of FANS f‐URS versus mini‐PCNL. They may discuss any questions with the local clinical team and consent during either their outpatient appointment or inpatient stay. Written informed consent will be obtained prior to any study‐specific procedures; those unable or unwilling to consent will be excluded. Upon receiving the participant's permission, their urologist will be informed of trial enrolment. Once consent and baseline assessments are completed, participants will be randomized to the FANS f‐URS or mini‐PCNL arm.

### 5.5 Randomization and allocation

Participants at respective institutions will be randomized in a 1:1 ratio to either the FANS f‐URS or mini‐PCNL arm, with each site enrolling 32 patients. Randomization will be performed centrally via a secure web‐based system at the time of enrolment, using computer‐generated permuted‐block sequences of varying sizes to maintain allocation concealment. Stratification by centre ensures balance across sites, and block sizes will be kept confidential. Investigators will obtain treatment assignments directly from the web application only after the patient's eligibility and consent have been confirmed.

### 5.6 Intervention

Two endourological approaches will be evaluated in this trial: (1) FANS f‐URS and (2) mini‐PCNL. Both interventions will follow the standardized protocols described in Section 5.7 to ensure consistency across all sites.

### 5.7 Operation protocol

A standardized operating methodology, approved by the principal investigator at each centre, will be implemented to ensure consistency across sites. Monthly monitoring visits will be conducted to verify adherence to the protocol and address any procedural deviations.

All patients will undergo non‐contrast CT and intravenous pyelogram preoperatively to confirm stone location and measure stone diameter, area and volume, as well as to assess the infundibulopelvic (IP) angle and infundibular length and width.^12^ Stone burden and density will be quantified using the same validated software at each centre. Standard perioperative antibiotic prophylaxis—either cefuroxime 1500 mg or levofloxacin 500 mg—will be administered 30 minutes before the procedure. Patients with a positive preoperative urine culture will receive tailored antibiotic therapy for 5–7 days based on sensitivity results prior to surgery.

#### 5.7.1 Endoscopic procedure

#### 5.7.2 FANS f‐URS

The procedure will be performed under general anaesthesia with the patient in the lithotomy position. A 5 Fr open‐ended ureteral catheter will be inserted into the ureter, followed by retrograde pyelography to evaluate the upper urinary tract. A 0.035/0.038‐in. guidewire will then be advanced into the renal pelvis. Depending on the patient's anatomy, ureteral condition and device availability, the surgeon will select a FANS‐sized 12/14 Fr, 11/13 Fr or 10/12 Fr. If even the smallest 10/12 Fr sheath cannot be advanced, a 6 Fr double‐J stent will be placed and the procedure scheduled two weeks later as staged f‐URS. All FANS f‐URS procedures will be performed using a 7.5 Fr single‐use digital flexible ureteroscope. Stones are fragmented using either a Holmium:YAG or Thulium fibre laser (≤30 W, 200 μm fibre), with continuous irrigation (50–150 ml/min) and suction (80–120 mmHg). Stone relocation will be allowed at the surgeon's discretion using baskets or other auxiliary tools if judged necessary for optimal stone clearance. Upon conclusion of the procedure, ureteral injuries will be visually assessed and classified according to the Traxer and Thomas endoscopic classification of ureteric injury,^13^ then both the FANS and scope will be withdrawn. A 6 Fr double‐J stent is placed for two weeks based on ureteral condition; routine Foley catheter placement is omitted.

#### 5.7.3 Mini‐PCNL

Under general anaesthesia, a 5 Fr ureteral catheter is placed cystoscopically and a 16 Fr Foley catheter is inserted for bladder drainage. For supine mini‐PCNL, the patient remains in the supine (Galdakao‐modified Valdivia) position; for prone mini‐PCNL, they are repositioned to prone following catheter placement. Percutaneous access will be achieved using an 18‐gauge coaxial needle to puncture the desired calyx under fluoroscopic or ultrasound guidance. Tract dilatation will be accomplished using fascial dilators up to 20 Fr. When multiple nephrostomy tracts are necessary to remove the stones, the same technique will be employed for each tract. Endoscopic combined intrarenal surgery (ECIRS) will not be allowed in this trial, to maintain protocol consistency and avoid confounding effects. Fragmentation of the stone burden will be accomplished using either a pneumatic lithotripter, Holmium:YAG or Thulium fibre laser. At the end of the procedure, a 6 Fr double‐J ureteral stent will be left in place. The decision to place a nephrostomy tube is made at the operating surgeon's discretion. Tubeless mPCNL will be selectively performed in patients who meet the following criteria: absence of significant residual stones, no visible perforations, no significant bleeding and no anticipated need for second‐session procedures.

### 5.8 Follow‐up and data collection

A low‐dose, non‐contrast CT scan with 2‐mm slices will be obtained within 72 hours post‐procedure to assess immediate stone‐free status, defined as no visible stones or only fragments ≤ 2 mm in the bone window. Routine blood tests and serum procalcitonin will be drawn within two hours of surgery to screen for infection, and haemoglobin will be compared between preoperative and two hours postoperative samples; any patient with a haemoglobin below 70 g/l will receive a blood transfusion.

In the FANS f‐URS group, patients with no significant discomfort may be discharged within 24 hours of surgery. In the mini‐PCNL group, if a nephrostomy tube was placed, it will be removed 1–3 days after the effluent becomes grossly clear, followed by patient discharge. All patients will have their 6 Fr double‐J stents removed at two weeks. Health‐related quality of life will be measured using the standardized Wisconsin Stone QoL questionnaire before surgery and again at one month.^14^ Stone composition will be analysed by infrared spectroscopy using a uniform methodology at each centre. At one month, a follow‐up low‐dose non‐contrast CT will be performed only in patients with residual fragments on the 72‐hour scan; those rendered stone‐free at 72 hours will not undergo further imaging.

Patients' characteristics and clinical outcomes will be recorded on a pre‐established case report form (CRFs). Stone size will be defined as the largest diameter of the stone. For multiple stones, the stone size will be defined as the cumulative sum of the maximal diameters of all stones. Grading of hydronephrosis will be evaluated according to the Onen hydronephrosis grading system.^15^ For the FANS f‐URS group, the operative time is characterized as the duration from the insertion of the endoscope per urethral to the completion of stent placement. For the mini‐PCNL group, the operation time is defined as from retrograde placement of ureteric catheter to the placement of nephrostomy tubing or removal of percutaneous access. Hospital stay will be rounded up to the nearest whole day and calculated from the day of surgery to the day of discharge.

Each recruited patient will have three CRFs completed by the research team at the recruiting site. A baseline CRF will be completed at the time of randomization. A treatment CRF will be completed following the randomized intervention (operation). The CRFs at 1‐month post‐operation will be completed and entered on‐site by the centre coordinators at the recruiting centres. Data on any additional interventions received and complications will be documented. Table 1 shows the schedule of outcome assessment and data collection.

**TABLE 1:** Schedule of enrolment, interventions and assessments.

Trial period	Screening (−4 wk to 0)	Allocation (0)	Post‐allocation	Close‑out (1 mo)
	Timepoint: −t1	Timepoint: 0	Timepoints: t1 = 72 h; t2 = 2 wk	Timepoint: t3 = 1 mo
ENROLMENT				
Eligibility screen	X			
Informed consent	X			
Preoperative CT & baseline labs	X			
Wisconsin Stone QoL (baseline)	X			
RANDOMIZATION & ALLOCATION				
Randomization via web system		X		
INTERVENTIONS				
FANS fURS or mini‑PCNL procedure		X		
ASSESSMENTS				
Immediate post‑op labs (Hb, PCT)			X	
Non‑contrast CT (2 mm slices)			X	
Pain VAS (24 h)			X	
Hospital stay (days)			X	
Double‑J stent removal			X	
Wisconsin Stone QoL (follow‑up)				X
Low‑dose CT if residual within 72 h				X
Complications (Clavien–Dindo grading)			X	X
Stone analysis (infrared spectra)			X	

QoL – quality of life; FANS‐fURS – flexible and navigable suction ureteral access sheath flexible ureteroscopy; mini‐PCNL – mini‐percutaneous nephrolithotomy; Hb – haemoglobin; PCT – procalcitonin; CT – computed tomography; VAS – visual analogue scale.

### 5.9 Blinding

Blinding of patients and operating surgeons is not feasible due to the obvious procedural differences between FANS f‐URS and mini‐PCNL. However, radiologists who review all postoperative CT scans will be blinded to treatment allocation. Clinical follow‐up assessments will be conducted by investigators who did not participate in the surgeries. Statistical analyses will also be performed in a blinded fashion: the analyst will remain unaware of group assignments, which will only be unmasked once data interpretation is complete.

### 5.10 Subject withdrawal

Participants will remain in the trial unless they choose to withdraw or are medically unable to continue. If a participant elects to withdraw, we will request permission to continue capturing relevant outcome data from their medical records. Any status changes short of complete withdrawal of consent—including loss to follow‐up—will be treated as ongoing participation, and every effort will be made to collect all scheduled outcome measures. In cases where the assigned procedure cannot be completed due to intraoperative factors (e.g., access failure in mini‐PCNL, failure of FANS placement or equipment malfunction [scope or sheath damage]), crossover to the alternative arm (FANS f‐URS ↔ mini‐PCNL) will be permitted. All crossover events will be clearly documented with reasons specified. For the primary analysis, crossover cases will be analysed according to the intention‐to‐treat (ITT) principle, while per‐protocol (PP) analyses will exclude these patients.

### 5.11 Sample size

Based on published literature, the immediate SFR for mini‐PCNL in 1–2 cm lower pole stones is approximately 85%; we therefore assume an immediate SFR of 85% for both the mini‐PCNL and FANS f‐URS arms^8^, ^17^, ^18^. A non‐inferiority margin of 8.5% has been selected, as an absolute reduction in SFR of up to 8.5% is deemed the maximum clinically acceptable loss of efficacy given the benefits of FANS f‐URS (less invasiveness, faster recovery and lower bleeding risk) and aligns with margins used in similar endourological trials.^8^ Using simulations conducted in Stata with an 80% power and an alpha level of 2.5%, the required sample size is 278 participants per group (totalling 556). To account for an anticipated 15% loss to follow‐up or withdrawal, we inflate each arm to 320 participants, yielding a final enrolment target of 640 patients.

### 5.12 Statistical analysis

Statistical analysis will be performed using SPSS software version 20.0. The primary analysis will follow the ITT principle, including all randomized participants in their assigned groups regardless of protocol adherence. A secondary PP analysis will include only those participants who complete the assigned intervention without major protocol violations, to assess the robustness of the ITT findings.

Primary and secondary outcomes will be analysed as follows: Continuous data that are normally distributed will be presented as mean ± standard deviation (x ± s), while non‐normally distributed data will be expressed as median (with interquartile ranges). For comparisons of normally distributed continuous data between groups, the independent samples t‐test will be used. The Mann–Whitney U test will be applied for comparisons of non‐normally distributed continuous data. Categorical data will be presented as frequency (rate) and analysed using the chi‐square test or Fisher's exact test. If multivariate analysis is required, multiple linear regression or logistic regression models will be employed, depending on whether the dependent variables are continuous or categorical. Statistical results will be reported as estimates with 95% confidence intervals (CIs), which will be compared to the pre‐specified non‐inferiority margin. Blinded statistical analyses will be performed, with the analyst unaware of group assignments. The group allocation code will remain concealed until the analyses and interpretation have been completed. A predefined subgroup analysis will stratify patients by IP angle (e.g. <30° vs. ≥30°) to evaluate whether anatomical variation influences comparative efficacy and safety. A mixed‐effects model or generalized estimating equations may be employed post hoc to adjust for potential clustering by study centre and assess the robustness of primary findings across sites.

### 5.13 Study flow chart

**FIGURE 1:**
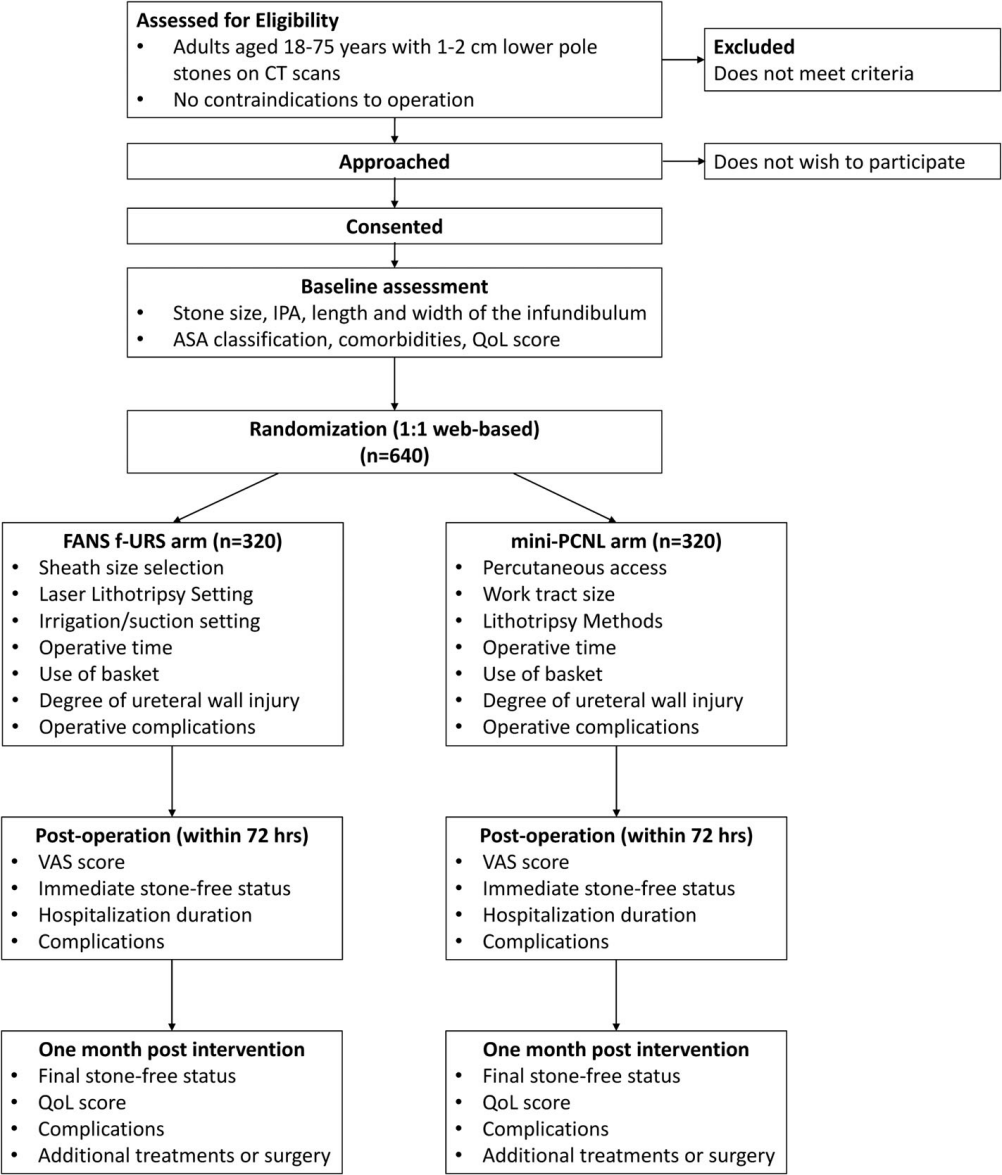
**Flowchart of the study process.** FANS = flexible and navigable suction ureteral access sheath; CT = computed tomography; IPA = infundibulopelvic angle; ASA = American Society of Anesthesiology; QoL = quality of life; VAS = visual analogue scale.

Please see the Figure 1.

### 5.14 Quality control

All participating centres are high‐volume urolithiasis centres, each performing over 300 FANS f‐URS and 200 mini‐PCNL procedures annually, ensuring timely enrolment of 640 eligible patients. Strict adherence to inclusion, exclusion and withdrawal criteria will be enforced at every site. Preoperative evaluations will be conducted by urologists with extensive stone management experience. A multidisciplinary oversight committee– comprising experts in urology, endourological surgery, biostatistics and clinical research– will guide trial conduct and data integrity.

A uniform, principal investigator–approved standard operating procedure will be adopted across centres to guarantee procedural consistency. Designated surgeons at each site (each with ≥200 FANS f‐URS and ≥100 mini‐PCNL cases per year) will perform all interventions. Monthly monitoring visits will audit compliance with the protocol, review CRFs and resolve any deviations.

All adverse events will be documented in the CRFs and reported immediately to the principal investigator. Each event will be categorized by expectedness, severity and relationship to the intervention. Serious or unexpected adverse events will be promptly reported to the central ethics committee. The principal investigator will conduct periodic cumulative safety reviews and convene investigator meetings as needed to address emerging concerns.

An independent committee of three senior urologists and one biostatistician will meet quarterly to review safety, enrolment, protocol adherence and unblinded interim efficacy using a Haybittle–Peto boundary. They may recommend continuation, modification or termination.

### 5.15 Data management

All trial data will be entered directly into a secure, web‐based electronic data capture (EDC) system by trained research coordinators. Each CRF field incorporates built‐in range and consistency checks—for example, stone diameter must be between 10 and 20 mm—to guard against obvious entry errors. Critical variables (primary outcome, complication occurrence, CT measurements) trigger automated validation alerts if values fall outside predefined limits. To further minimize transcription errors, 10% of participants' records at each site will be randomly selected each month for independent double data entry by a second coordinator; any discrepancies will be reconciled against source documents.

Data are encrypted in transit (TLS/SSL) and at rest on our centralized server, which is protected by institutional firewalls and backed up nightly to an off‐site encrypted archive. Access is managed via role‐based permissions: site coordinators may enter and correct data for their own centre, while the central data manager and independent biostatistician have privileges to review, query and export the full dataset for analysis. A comprehensive audit trail logs every create, update and delete action—recording the timestamp, user ID and reason for modification.

All electronic data and de‐identified source documents will be retained for a minimum of 10 years after study completion, in accordance with institutional and regulatory requirements. A detailed Data Management Plan—detailing field definitions, validation rules, backup schedules and disaster recovery procedures—is available upon request from the coordinating centre.

### 5.16 Ethics and dissemination

This multicentre trial will be conducted in accordance with the Declaration of Helsinki and ICH‐GCP guidelines. The Ethics Committee of the lead site—the Institutional Review Board of The First Affiliated Hospital of Guangzhou Medical University—has performed a central ethical review and granted approval. Participating centres will adopt this approval and conduct a local feasibility review (focusing on investigator qualifications and site capabilities) via an expedited meeting or board review. Consent will be obtained in person by trained study nurses in the outpatient clinic or inpatient ward. All CRFs are de‐identified; identifying keys are held separately on encrypted, access‐controlled servers for 10 years post‐trial.

Upon completion, the study results will be submitted for publication in peer‐reviewed international journals and presented at national and international urology conferences. Authorship follows ICMJE guidelines. De‐identified participant‐level data and statistical code will be made available upon reasonable request to the corresponding author after publication.

## 6 DISCUSSION

This international, multicentre, randomized non‐inferiority trial is designed to address a critical gap in the management of 1–2 cm lower pole renal stones: whether FANS f‐URS can achieve SFRs comparable to mini‐PCNL, while offering the well‐documented benefits of a less invasive approach. By leveraging the combined expertise of 20 high‐volume centres, our protocol ensures both rapid patient accrual and procedural consistency, thereby enhancing the statistical power and generalizability of our findings.

The primary strength of this study lies in its rigorous non‐inferiority design with a clinically meaningful 8.5% margin, informed by expert consensus and precedent in urolithiasis research. Blinded assessment of imaging outcomes and centralized randomization further minimize bias, while a robust per‐protocol and intention‐to‐treat analysis plan will verify the stability of our conclusions. Inclusion of detailed secondary endpoints—operative time, hospital stay, pain scores, complications and quality of life—will furnish a comprehensive view of each technique's risk–benefit profile.

Nonetheless, several limitations merit consideration. First, neither patients nor surgeons can be blinded to treatment allocation, which may introduce performance or reporting bias; however, the blinding of radiologic reviewers and outcome assessors should mitigate this risk. Second, despite uniform training and monitoring, subtle differences in surgical technique or postoperative care among centres may contribute to outcome variability. Predefined subgroup analyses by IP angle and centre‐effect adjustments will help elucidate and account for these factors. Third, the non‐inferiority margin, while carefully chosen, remains an arbitrary threshold; should the observed difference approach this boundary, interpretation will require careful clinical judgement.

This trial does not incorporate a formal cost‐effectiveness or health‐economic analysis. The main reason is that reimbursement standards and device costs vary substantially across participating centres and countries, making accurate and comparable cost assessment unfeasible within the current study design. To partially address this, we will document all supplementary device use as part of the trial records, although a comprehensive economic evaluation remains outside the scope of this study and should be pursued in future research.

If FANS f‐URS proves non‐inferior to mini‐PCNL in SFR and demonstrates clear advantages in safety, recovery or patient‐reported outcomes, its adoption could potentially guide recommendations toward a more minimally invasive standard for 1–2 cm lower pole stones. Conversely, should mini‐PCNL retain superior efficacy beyond our margin, these data will reaffirm its role as the gold‐standard treatment. Either outcome will directly inform clinical practice, support evidence‐based guideline updates and ultimately improve patient care.

In conclusion, this trial will generate high‐quality evidence to guide the optimal endourological management of 1–2 cm lower pole stones. Its multicentre scope, rigorous methodology and focus on both efficacy and patient‐centered outcomes position it to make a definitive contribution to the field of urolithiasis.

## 7 PLAN OF PROGRESS

Patient recruitment will start in August 2025 and continue through July 2026. Follow‐up will be conducted according to the protocol. We expect the final 1‐month follow‐up to be completed by August 2026. Upon completion of the last follow‐up, we will perform statistical analyses of data, report and publish the results in a timely manner.

## AUTHOR CONTRIBUTIONS

Guohua Zeng had full access to all the data in the study and takes responsibility for the integrity of the data and the accuracy of the data analysis.

*Study concept and design: Guohua Zeng, Wei Zhu*, Steffi Kar Kei Yuen *and* Vineet Gauhar.

Acquisition of data: Wei Zhu, Jinghua Zhong, Kefeng Wang, Guangming Yin, Chi‐Ho Leung, Nariman Gadzhiev, Mehmet Ilker Gökce, Jaisukh Kalathia, Guangyuan Zhang, Gang Wu, Mingyong Li, Li Fang, Shaohua Zeng, Peide Bai, Ji Li, Zhenhua Zhao, Ming Xi, Xin Mai, Xianzhong Duan, Kehua Jiang, Jianwei Cao, Xianghu Meng, Shusheng Liu, Aram Aloyan, Peng Yuan, Xiaorui Zhu, Ting Huang, Jianhui Xing, Jingzeng Du, Wenqi Wu, Jinxiang Ma, Wen Zhong, Zhijian Zhao, Yongda Liu, Chi Fai Ng, Jean de la Rosette, Shiyong Qi, Steffi Kar Kei Yuen.

Analysis and interpretation of data: Chi‐Ho Leung, Jinxiang Ma.

Drafting of the manuscript: Wei Zhu and Steffi Kar Kei Yuen.

Critical revision of the manuscript for important intellectual content: Vineet Gauhar. Statistical analysis: Chi‐Ho Leung, Jinxiang Ma.

Obtaining funding: Guohua Zeng.

Administrative, technical, or material support: Jinxiang Ma, Vineet Gauhar, Steffi Kar Kei Yuen, Jean de la Rosette.

Other: None.

## CONFLICT OF INTEREST STATEMENT

On behalf of all authors, Guohua Zeng declares a potential conflict of interest, as MacroLux Medical Technology Co., Ltd., the manufacturer of the 7.5 Fr single‐use digital flexible ureteroscope, provided flexible ureteroscopes to centres as an in‐kind contribution. No other payments were made to the institutions, hospitals or any of the authors related to this study. The only support provided by the company was the in‐kind contribution of the single‐use digital flexible ureteroscope.
